# The role of interleukin-1 in neuroinflammation and Alzheimer disease: an evolving perspective

**DOI:** 10.1186/1742-2094-5-7

**Published:** 2008-02-26

**Authors:** Solomon S Shaftel, W Sue T Griffin, M Kerry O'Banion

**Affiliations:** 1 Department of Neurobiology and Anatomy, University of Rochester School of Medicine and Dentistry, Rochester, New York, USA; 2 Department of Geriatrics, University of Arkansas for Medical Sciences, Little Rock, Arkansas, USA; 3 Department of Neurology, University of Rochester School of Medicine and Dentistry, Rochester, New York, USA

## Abstract

Elevation of the proinflammatory cytokine Interleukin-1 (IL-1) is an integral part of the local tissue reaction to central nervous system (CNS) insult. The discovery of increased IL-1 levels in patients following acute injury and in chronic neurodegenerative disease laid the foundation for two decades of research that has provided important details regarding IL-1's biology and function in the CNS. IL-1 elevation is now recognized as a critical component of the brain's patterned response to insults, termed neuroinflammation, and of leukocyte recruitment to the CNS. These processes are believed to underlie IL-1's function in the setting of acute brain injury, where it has been ascribed potential roles in repair as well as in exacerbation of damage. Explorations of IL-1's role in chronic neurodegenerative disease have mainly focused on Alzheimer disease (AD), where indirect evidence has implicated it in disease pathogenesis. However, recent observations in animal models challenge earlier assumptions that IL-1 elevation and resulting neuroinflammatory processes play a purely detrimental role in AD, and prompt a need for new characterizations of IL-1 function. Potentially adaptive functions of IL-1 elevation in AD warrant further mechanistic studies, and provide evidence that enhancement of these effects may help to alleviate the pathologic burden of disease.

## Introduction

Interleukin-1 (IL-1) comprises a pleiotropic cytokine family capable of numerous actions in the central nervous system (CNS). IL-1 classically refers to a 17 kilodalton (kDa) polypeptide existing in two distinct isoforms, IL-1α and IL-1β, although other members of the IL-1 family have recently been proposed [1]. Although IL-1α and IL-1β are encoded by separate genes sharing some sequence homology, they elicit similar biological actions. In addition to these two IL-1 receptor agonists, a native IL-1 receptor antagonist (IL-1ra) also maps to the IL-1 gene cluster on human chromosome two. All three proteins are produced as precursors, of which pro-IL-1α and pro-IL-1ra possess biological activity. Pro-IL-1β, however, requires cleavage by caspase-1 (IL-1β converting enzyme, ICE) to become biologically active. Details about the structure and regulation of these family members, as well as information about many of their actions can be found in recent reviews [2-4].

All known actions of IL-1 are mediated by a single biologically active 80 kDa cell surface receptor, the type I IL-1 receptor (IL-1RI) [5]. IL-1R1 is expressed throughout the rodent brain, with levels generally highest in neuronal rich areas including the dentate gyrus, the pyramidal cell layer of the hippocampus, and the hypothalamus [6,7]. Binding of IL-1 agonists to IL-1R1 requires association with an accessory protein to elicit downstream signal transduction that includes activation of nuclear factor-kappa B (NFκB) and mitogen-activated protein (MAP) kinase pathways [8,9]. While all known biological functions of IL-1 are attributable to IL-1 interactions with IL-1R1, some studies suggest that alternate functional IL-1 receptors may exist in the CNS [10,11].

The evolutionary importance of IL-1 activity within the brain is highlighted by the presence of two distinct endogenous regulatory pathways. IL-1ra is a competitive antagonist of IL-1R1 that selectively binds, but fails to trigger receptor association with the accessory protein resulting in blockade of all known actions of IL-1. A second 68 kDa receptor, the type II IL-1 receptor (IL-1RII), may serve as a decoy as it binds all IL-1 ligands but lacks an intracellular domain and has no demonstrated signaling function [12]. Further description of the IL-1 regulatory pathways can be found in two recent comprehensive reviews [1,13].

### IL-1 actions within the CNS

IL-1 was the first cytokine identified with actions on the brain [14,15]. Its ability to elicit fever after peripheral administration led to early descriptions of IL-1 as the "endogenous pyrogen". The research that followed has implicated IL-1 in a diverse array of physiologic and pathologic processes within the mammalian CNS, and has earned IL-1 status as a prototypic pro-inflammatory cytokine [13,16,17]. Generally speaking, the actions of IL-1 in the CNS are attributed to either responses of the neuroendocrine system or the local tissue microenvironment.

In response to homeostatic threats in mammals, increased IL-1 levels activate the hypothalamo-pituitary-adrenal (HPA) axis and are central to elicitation of sickness behaviors. The downstream effects of this neuroendocrine system stimulation likely underlie the ability of IL-1 to modulate processes such as appetite, body temperature, epilepsy, and sleep/wake cycles in mammals [16,18-20]. This review will focus on IL-1 as a key regulator of local tissue responses to injury and disease in the CNS, with emphasis on its role in neuroinflammation.

### Expression of IL-1 in injury and disease

Initial evidence that IL-1 may play a key role in local brain tissue reactions came from demonstrations of elevated IL-1 expression in a diverse array of CNS diseases. In humans, IL-1 is elevated in brain tissue and cerebrospinal fluid (CSF) from patients who succumbed to brain injury or stroke [21]. This pattern of expression was further extended to animal models of CNS injury where parenchymal IL-1 mRNA and protein levels are elevated in experimental models of ischemia, excitotoxicity, infection and traumatic brain injury in rodents. While IL-1α and IL-1β are barely detectable in either normal human or rodent brain, they are rapidly induced within minutes of acute experimental injuries [reviewed in [1,3,22]].

In addition to initial demonstrations of IL-1 elevations following acute injuries, similar observations have been made in a number of chronic neurodegenerative disorders. IL-1 elevations have been reported within brain lesions from patients with Alzheimer's disease (AD), Multiple Sclerosis (MS), Down's Syndrome and HIV-associated dementia [17,23-25]. Furthermore, increased IL-1 has been detected in CSF samples in MS, Parkinson's and Creutzfeldt-Jakob disease (CJD) [26-28]. These findings have since been reproduced in corresponding animal models of disease for AD, MS and CJD [29-33].

### Sources and targets of IL-1 expression

IL-1 is both expressed by and targeted to many different cell types within the CNS. Microglia express ICE, and in response to injury are thought to produce both the initial burst and highest levels of IL-1 production [34]. IL-1 can also be produced by astrocytes, endothelial cells, infiltrating leukocytes, neurons and oligodendrocytes [4,35,36]. In turn, IL-1 can feed back on its original cellular sources but is thought to exert its primary actions on microglia, astrocytes and endothelial cells [2].

Neuroglia and endothelial cells produce a myriad of signaling molecules in response to IL-1 stimulation. These include pro-inflammatory cytokines, chemokines, adhesion molecules, prostaglandins, reactive oxygen species, nitric oxide, and matrix metalloproteases. Notably, IL-1 induces expression of the pro-inflammatory cytokines tumor necrosis factor alpha (TNF-α) and Interleukin-6 (IL-6) as well as the enzyme cyclooxygenase-2 (COX-2) in both astrocytes and microglia in culture [4,37,38]. These inflammatory mediators have been implicated in the propagation of a number of CNS injuries and diseases [39].

### IL-1β and neuroinflammation

Neuroinflammation is traditionally defined as the brain's innate immune response to injury. The hallmarks of a neuroinflammatory response are phenotypic glial activation and de novo production of immune signaling molecules. Both astrocytes and microglia undergo cellular hypertrophy with increased expression of cell-surface immune modulatory proteins, including those of the major histocompatibility complex (MHC). These changes are accompanied by increased synthesis and release of pro-inflammatory cytokines and chemokines.

IL-1β is intimately involved in elaboration of acute neuroinflammatory processes in vivo. Exposure of the rodent brain to IL-1β elicits rapid, robust activation of both astrocytes and microglia. In addition, single bolus injection or parenchymal expression of IL-1β in rodents increases expression of pro-inflammatory cytokines, leukocyte chemotactic chemokines, cell surface adhesion molecules, cyclooxygenases and matrix metalloproteases within the brain parenchyma [40-45]. Importantly, IL-1β is capable of triggering further increases in it's own expression as evidenced by murine IL-1β induction following human IL-1β administration or expression in the brain [45,46]. By feeding back upon itself, small localized elevations in IL-1 may be sufficient to drive potent neuroinflammatory changes in the brain. Further evidence for a central role of IL-1 in neuroinflammation has been provided in IL-1R1 knockout mice where lack of IL-1 signaling in the setting of penetrating brain injury causes dramatic attenuation in microglial and astrocytic activation as well as IL-6 and COX-2 production [47,48].

### IL-1 and leukocyte recruitment

In addition to elaboration of a robust neuroinflammatory response in the CNS, IL-1 elevations have been implicated in trafficking of peripheral immune cells to the brain. The normal, healthy CNS has relatively few, if any leukocytes [49]. However, cellular populations within the brain can quickly change following injury. The inflammatory response that follows CNS insults such as infection or injuries caused by trauma or ischemia often features rapid infiltration of leukocytes into the brain [50-52].

IL-1β expression is a powerful stimulus for leukocyte recruitment to the CNS. Either single bolus injection or localized expression of IL-1β within the rodent brain is capable of overriding the brain's intrinsic resistance to leukocyte recruitment, resulting in rapid cellular infiltration of the parenchyma. Cell types recruited include neutrophils, CD4+ and CD8+ T-cells, dendritic cells, and cells of the monocyte lineage [40,43,44,46,53,54]. This leukocyte infiltration is dependent on IL-1R1, and can be significantly reduced following administration of IL-1ra [44,53,55,56]. The ability of IL-1β to drive enhanced expression of monocyte chemoattractant protein-1 (MCP-1; CCL2) by astrocytes and intercellular adhesion molecule-1 (ICAM-1) on vascular endothelial cells within the brain is thought to facilitate parenchymal cellular recruitment [40,44]. Indeed, adenoviral overexpression of IL-1ra following experimental ischemia reduces ICAM-1 expression and leukocyte infiltration of the brain [57].

Neutrophils play a vital role in the innate arm of the immune response that rapidly develops at sites of injury and infection. While primarily recognized for destruction of invading pathogens, neutrophils can also shape immune responses [58]. The chemokines of the ELR^+ ^CXC family are potent stimuli for the recruitment and activation of neutrophils in peripheral and CNS inflammatory responses, and are upregulated by acute IL-1β stimuli [59-63]. In mice, the most potent and well defined members of this family are keratinocyte-derived chemokine (KC, CXCL1) and macrophage inflammatory protein 2 (MIP-2, CXCL2) which are thought to signal exclusively through the CXCR2 receptor [50,60,63-65]. Interestingly, chronic IL-1β expression can serve as a long-lasting stimulus for MIP-2 and KC induction as well as neutrophil recruitment to the brain. Using a mouse model of chronic hippocampal human IL-1β overexpression, we observed neutrophil infiltration of the brain parenchyma months after initiation of transgene activation. This neutrophil recruitment appeared to be dependent on signaling through the CXCR2 receptor, as it was absent in CXCR2 knockout mice [44].

Leukocyte recruitment to the CNS is highly restricted by presence of the blood-brain barrier (BBB), which is credited for the virtual absence of leukocytes within the healthy brain parenchyma [66]. Although not thought to be necessary nor sufficient for leukocyte infiltration of the CNS, breakdown of the BBB is believed to potentiate cellular recruitment to the brain [49]. Experimentally induced elevations of IL-1β levels in the brain cause disruptions in the BBB, which may underlie its effectiveness as a leukocyte recruitment stimulus [41,43,44,54]. Neutrophils have been implicated in mediating this effect based on a study in rats where administration of anti-CINC-1 (CXCL1) neutralizing antibodies attenuated neutrophil recruitment and BBB breakdown downstream of intracerebral IL-1β injections [41]. However, recent work in our laboratory indicates that IL-1β can influence BBB integrity even in the relative absence of neutrophil recruitment. More specifically, we observed significant leakage of albumin bound Evan's blue dye into the brain parenchyma of mice engineered to chronically express human IL-1β in a sustained manner, which was not altered in animals lacking the CXCR2 receptor [44]. The precise mechanisms of IL-1β mediated changes in BBB permeability remain unclear. Further studies are needed to elucidate this phenomenon, as it may be important in the pathogenesis of CNS injury and disease.

### Learning, memory, and IL-1

In addition to its role in elaboration of neuroinflammation and leukocyte recruitment, local expression of IL-1 has been implicated in impairment of hippocampal dependent memory processing [67]. IL-1β activity is thought to be closely tied to the process of memory consolidation based on demonstrations of increased IL-1β expression in vitro and in vivo during long-term potentiation (LTP), a process that is believed to underlie hippocampal dependent learning and memory processes in mammals [68]. At sufficient concentrations, IL-1β is capable of blocking hippocampal LTP [69]. Also, injection of lipopolysaccharide (LPS), a potent inducer of IL-1β expression by microglia, into the rat hippocampus results in learning and memory deficits [70]. Conversely, blockade of IL-1R1 in rats using adenoviral expressed IL-1ra leads to facilitation of short and long-term memory in an inhibitory avoidance task [71]. Based on these data, it is feasible that IL-1 elevations, as occur following CNS injuries and during neurodegenerative disease, might lead to impairments in learning and memory. This may help explain the prominent memory deficits characteristic of AD and HIV-associated dementia.

### The contribution of IL-1 to acute CNS injury

The elevation of IL-1 expression following a diverse array of acute brain injuries coupled with its ability to elicit diverse inflammatory changes as previously discussed, suggests that it may contribute to the pathogenesis of CNS injury. Indeed, data from head injury victims as well as in acute experimental brain injury paradigms has provided strong evidence in support of this [21]. To date most studies have focused on the CNS actions of IL-1β, rather than IL-1α, based on its more rapid induction following injury [16]. A number of studies have demonstrated that administration of IL-1β concurrent with experimental ischemia is capable of exacerbating injury [1,72]. Conversely, ischemic damage is greatly reduced by knockout of IL-1α and IL-1β, administration of IL-1 blocking antibodies, or disruption of interleukin-1 converting enzyme function [2,73,74]. Interestingly, there is some evidence that IL-1 may influence ischemic injury independent of IL-1R1 [11]. In traumatic brain injury, neuronal damage is similarly reduced by administration of IL-1ra [75]. Based on the ability of exogenously administered IL-1ra to attenuate experimental ischemic injury and cross the BBB, therapy with human IL-1ra has been investigated in a Stage II clinical trial of stroke [76-78].

Increased levels of endogenous IL-1ra and IL-1RII are likely to be important mechanisms for regulation of IL-1 activity following brain injury. IL-1ra is rapidly induced following experimental injury, and blockade of endogenous production leads to exacerbation of neurotoxicity following ischemic injury in rodents [74,79]. Increased expression of IL-1RII, the biologically inactive "decoy" IL-1 receptor, has been demonstrated following injection of IL-1β into the brain parenchyma and may also serve to limit the biological function of IL-1 [12].

In addition to data implicating IL-1 in exacerbation of acute injury, other studies have provided evidence for beneficial effects of IL-1 signaling within the brain. IL-1 has been associated with neuroprotective mechanisms in rodent primary neuron cultures which may be mediated in part by production of survival signals such as nerve growth factor (NGF) [80-82]. IL-1 signaling has also been implicated in re-myelination of the CNS after cuprizone demyelination injury, which may be due in part to IL-1 mediated stimulation of oligodendrocyte proliferation [35,83]. Finally, absence of IL-1R1 has been associated with deficiencies in hippocampal dependent spatial learning but it is unclear if this is a result of specific beneficial influences of IL-1 on the intact nervous system or represents developmental alterations in the knockout mouse [84].

### Mechanisms of IL-1 induced neuronal damage

Due to its pleiotropic actions in the brain it has been difficult to pinpoint the mechanisms by which IL-1 exacerbates acute CNS injuries. In general, it does not appear that IL-1β is capable of triggering direct neurotoxicity when administered to the healthy adult rodent brain [16]. For example, chronic hippocampal overexpression of human IL-1β in mice does not engender overt neurotoxicity or changes in measures of neuronal integrity [44]. In rats, IL-1β induced neurotoxicity has been reported following single bolus injection or adenoviral mediated expression of IL-1β in the substantia nigra or hippocampus, respectively [46,85,86]. However, these studies in rats may have been confounded by tissue injury resulting from parenchymal injections, viral induced inflammation, or the use of non-physiologic doses of IL-1β. It is also possible that the capacity of IL-1β to mediate direct neurotoxicity may be a species-specific phenomenon. In vitro, IL-1β exposure does not affect the viability of pure mouse or rat neuronal cultures and can reduce excitotoxin induced neurotoxicity [81,82]. However, in rat mixed glial/neuronal cultures IL-1β has been reported to cause neurotoxicity through downstream free radical release [87].

Neutrophils have emerged as possible perpetrators of neuronal damage following acute brain insults downstream of IL-1β elevations [40,41,43,53]. Neutrophils are rapidly recruited following CNS injury, present at the time of neuronal death, and can trigger tissue damage through generation of toxic free radicals, proteolytic enzymes and pro-inflammatory cytokines such as IL-1β and TNF-α [52]. In vitro, co-cultures of rat neutrophils and primary hippocampal neurons demonstrate neurotoxicity in the absence of physiologic insults as well as exacerbation of kainic acid excitotoxicity [88]. In vivo, constitutive CNS overexpression of the chemokine KC results in striking recruitment of neutrophils to multiple brain regions and early neurological demise [89]. Depleting neutrophils or limiting their infiltration through ICAM-1 gene ablation attenuates experimental ischemic injury [52,90]. Despite these observations, recruitment of neutrophils does not appear to be sufficient for neurotoxicity. This point is supported by recent work from our laboratory where no evidence of overt neurotoxicity, synaptic damage, or loss of acetylcholine fibers was observed after 2 weeks or 2 months of sustained IL-1β induced neuroinflammation accompanied by prominent neutrophil infiltrates [44]. However, this study was limited to neuroinflammation in the dentate gyrus in the absence of concomitant injury, and it is possible that neurons in other brain regions may be more susceptible to neutrophil-mediated effects.

### Neuroinflammation and AD

Neuroinflammation is now recognized as a fundamental response of the CNS not only to acute injury, but also to chronic neurodegenerative disease. This is perhaps no better demonstrated than in AD, where the severity of the neuroinflammatory response parallels the disease course [91,92]. Neuroinflammation can be considered part of a characteristic pathologic triad of AD that includes amyloid plaques and neurofibrillary tangles. The neuroinflammatory phenotype in AD is characterized by robust activation of microglia and astrocytes in the vicinity of plaques, endogenous expression of pro-inflammatory cytokines, cell adhesion molecules, and chemokines [17,93-96]. These changes are thought to result from glial reaction to events related to ongoing deposition of amyloid β (Aβ) [97-99].

Epidemiological studies of nonsteroidal anti-inflammatory drug (NSAID) users lent credence to initial hypotheses as to the role of neuroinflammation in AD. Early observations among identical twins discordant for AD onset showing that those receiving anti-inflammatory therapy had delays in disease onset provided strong evidence for a detrimental role of neuroinflammation in AD pathogenesis [100]. Soon thereafter, additional case-based and longitudinal epidemiologic studies confirmed these findings, and demonstrated substantial reductions in disease incidence among patients on long-term regimens of NSAIDs [101-103]. In fact, recent meta-analysis has revealed as much as a 50% reduction in the risk of acquiring disease among chronic NSAID users [104]. This data led to a surge of research activity directed at elucidating the role of inflammation in AD and other chronic neurodegenerative disorders in hopes of designing new effective anti-inflammatory therapies for disease.

Unfortunately, clinical trials of traditional anti-inflammatory agents for treatment of patients with AD have failed to demonstrate efficacy [105-110]. Also, the only trial designed to directly address the hypothesis that chronic NSAID use can prevent AD in cognitively normal subjects failed to demonstrate a protective effect [111]. Drawing conclusions from these studies is complicated by the selection and dose of NSAIDs used, length of trials and overall designs [112]. Furthermore, the patient populations in these trials differ from their epidemiologic counterparts as patients in the latter were being treated with NSAIDs for inflammatory disorders which may have modified their risk of acquiring AD. Overall, the stark contrast between these results and the early epidemiological studies of chronic NSAID users suggests a complex role for neuroinflammation in AD.

Transgenic mouse models of AD harboring familial amyloid precursor protein (APP) mutations have recapitulated in part the intimate relationships between neuroinflammation and disease pathogenesis. In APPV717F mice astrocyte activation was evident before plaques were detected, and in the Tg2576 mouse model increased microglial density was observed in regions of Aβ deposits [113,114]. Similar neuroinflammatory changes have been observed in other murine models of AD, including the APP/PS1 double transgenic and APP/PS1/Tau triple transgenic (3xTg-AD) mice [115-117]. Finally, NSAID administration reduced both plaque pathology and neuroinflammatory measures in the Tg2576 mice [32].

### Possible roles for IL-1 in AD

IL-1 elevations became closely tied to AD pathogenesis soon after the discovery of prominent neuroinflammation in AD brain. Increased IL-1 expression in reactive microglia surrounding amyloid plaques provided the initial indication that IL-1 may be associated with AD pathogenesis [17]. Since that time, IL-1β elevations have been detected in the brains of aged AD mouse models and plaque associated microglia [31,32]. Microglial IL-1 activity was later tied to the evolution of plaques in AD [118]. In Down's syndrome, where patients are predisposed to AD neuropathological changes, IL-1 elevation and neuroinflammation precede by years the formation of plaques [119]. Additionally, specific polymorphisms in the IL-1α and IL-1β genetic loci were shown to be associated with increased disease risk in certain patient populations [reviewed in [120,121]]. These associations, in addition to observations of IL-1 elevations in AD patients, provided the key evidence for a central role of IL-1 in disease pathogenesis. However, recent meta-analysis has not supported a clear association between IL-1 genetic loci and AD when the data is examined as a whole [122].

IL-1 has been implicated in both the initiation and propagation of neuroinflammatory changes seen in AD through several lines of indirect evidence [reviewed in [39,123]]. Obviously, the known ability of IL-1 to drive robust neuroinflammatory changes in the acute setting adds to its attractiveness as a prime candidate for these actions. In AD, neuronal injury or insults including amyloid deposition may trigger a self-propagating cytokine cycle, which when chronically induced initiates a vicious feedback loop of continuing IL-1β elevation promoting further neuronal and synaptic dysfunction and Aβ plaque accumulation [99]. In support of this idea, cultured human monocytes and mouse microglia produce IL-1β in response to Aβ exposure or, to a greater extent, to secreted fragments of β-APP [124,125]. In other tissue culture studies IL-1 has been shown to increase β-APP mRNA expression, translation, and processing perhaps through enhanced gamma secretase activity [126-130]. In addition, IL-1β injection into rat brain results in elevation of β-APP [131]. Furthermore, IL-1ra knockout mice demonstrate enhanced human amyloid-beta induced neuropathology, suggesting the unopposed action of IL-1 as a likely culprit [132]. Although these findings are largely in vitro based, correlations in Alzheimer and control patients support the idea that these basic mechanisms occur in the disease itself.

In addition to association with modulation of β-APP processing, IL-1 activity has been tied to exacerbation of neurofibrillary tangles. Implantation of slow release IL-1β pellets in rats led to microglial activation and MAPK-p38-mediated hyperphosphorylation of tau protein, which is thought to contribute to microtubule destabilization and ultimately to formation of neurofibrillary tangles [133,134]. Analogous studies have been reported in cortical neuron cultures [135]. Microglial activation may be responsible for these effects as suggested by a recent study of synapse loss in a mouse tauopathy model [136]. Further support for IL-1β mediated activation of microglia and resultant tau hyperphosphorylation has been indirectly provided in the 3xTg-AD mouse model which expresses human mutated forms of tau, presenilin-1 and APP, and is characterized by both Aβ and tau pathology. Intraperitoneal LPS injections elicited significant IL-1β induction, microglial activation and accelerated the time course of tau hyperphosphorylation. Interestingly, LPS activity did not affect APP processing or Aβ deposition [137].

### A beneficial role for neuroinflammation in AD

While epidemiologic and experimental studies lend strong support for neuroinflammatory responses as drivers of AD pathogenesis, recent work also supports a beneficial role for such reactions [reviewed in [98]]. A number of reports have provided evidence that activation of microglia and their subsequent degradation of amyloid plaques may underlie this phenomenon. Direct injection of LPS into the CNS, which drives IL-1β synthesis and robust microglial activation, yields reductions of Aβ levels and plaque load [138-140]. Microglial activation has also been suggested to underlie enhanced plaque clearance in other transgenic AD models following treatment with either a modified nitric oxide-releasing NSAID (NCX-2216) or transforming growth factor beta overexpression (TGF-β) [141,142]. Conversely, inhibition of microglial activation with minocycline can increase Aβ deposition [143]. Although these reports and others suggest a beneficial role of microglial activation in mouse models of AD, it is worth noting that LPS stimulated neuroinflammation has also been associated with increased Aβ deposition [144,145].

Microglial activation is thought to reduce plaque burden through phagocytosis of Aβ peptides. There is substantial evidence for plaque associated microglia as phagocytosing scavengers of amyloid in vivo [146]. Indeed, the efficacy of antibody-mediated plaque clearance in AD mouse models appears in part to be mediated by enhanced phagocytosis by microglia [147,148]. In support of this, interference with microglial activation during Aβ immunotherapy reduced clearance of fibrillar deposits [149].

Infiltration of peripheral immune cells into sites of pathology, though not reported in AD itself, may enhance the beneficial effects of microglial mediated plaque clearance. It has been convincingly demonstrated, using green fluorescent protein (GFP) expressing bone marrow transplants in AD mouse models, that a proportion of activated microglia adjacent to amyloid plaques are in fact recruited from bone marrow-derived myeloid populations [150,151]. LPS administration enhances the seeding of bone marrow-derived myeloid cells in the brain which may in part explain its ability to enhance amyloid plaque clearance [150]. Selective elimination of bone marrow-derived myeloid cells exacerbates plaque pathology in AD models, and providing strong evidence that this microglial sub-population is efficient at degrading plaque [151,152]. Interestingly, an infiltrative cell type with dendritic cell-like characteristics appears to be particularly important in this process [153]. Taken altogether, these results suggest that the beneficial effects of neuroinflammation may in part result from increased recruitment of bone marrow-derived cells to the brain.

### A new view of IL-1's role in AD?

Since the original observations of IL-1 elevation in AD two decades ago, a body of evidence has implicated this proinflammatory cytokine as contributing to the pathogenic processes characteristic of disease. Much of this data has relied on indirect evidence and extrapolation from studies in tissue culture and acute injury paradigms. As is the case for neuroinflammation, contemporary studies in animal models of AD are challenging our original assumptions as to the role of IL-1 in AD.

Substantial support for an adaptive role of IL-1 elevation comes from a model developed in our laboratory to specifically address the function of chronic IL-1 driven neuroinflammation in AD pathology. When IL-1β was chronically overexpressed in the hippocampus of APP/PS1 transgenic animals, we witnessed a surprising reduction in both plaque pathology and insoluble amyloid peptide without evidence of effects on Aβ processing or APP expression. There was also a striking increase in numbers of plaque associated myeloid cells, suggesting enhanced phagocytosis of Aβ by microglia or infiltrating myeloid cells [45]. A limitation of this study is the use of a heterologous APP promoter, as this does not allow for interplay between inflammation and the APP promoter as discussed earlier. However, IL-1β overexpression was not associated with increased levels of murine APP mRNA [45].

The initial stimulus for elevation of IL-1 in AD is likely the result of exposure of microglia to injured neurons, β-APP, and its cleavage product Aβ as has been demonstrated both in vitro and in vivo [125,151]. Microglia chronically exposed to these stimuli during the course of disease likely mount sustained elevations in IL-1 and drive a self-perpetuating cycle of IL-1 overexpression in the brain parenchyma leading to chronic neuroinflammation [45]. As highlighted above, IL-1 elevation may potentiate plaque degradation by enhancing microglial activation and phagocytic activity, as well as seeding of peripheral phagocytic cells to areas of plaque deposition [44]. Despite this evidence pointing toward an important function of IL-1 in AD pathogenesis, a recent study in the Tg2576 AD mouse model failed to detect any influence of IL-1R1 knockout on either Aβ deposition or the efficacy of passive immunotherapy [154]. However, these results must be interpreted with caution as IL-1R1 knockout animals may be affected by compensatory changes during development.

**Figure 1:**
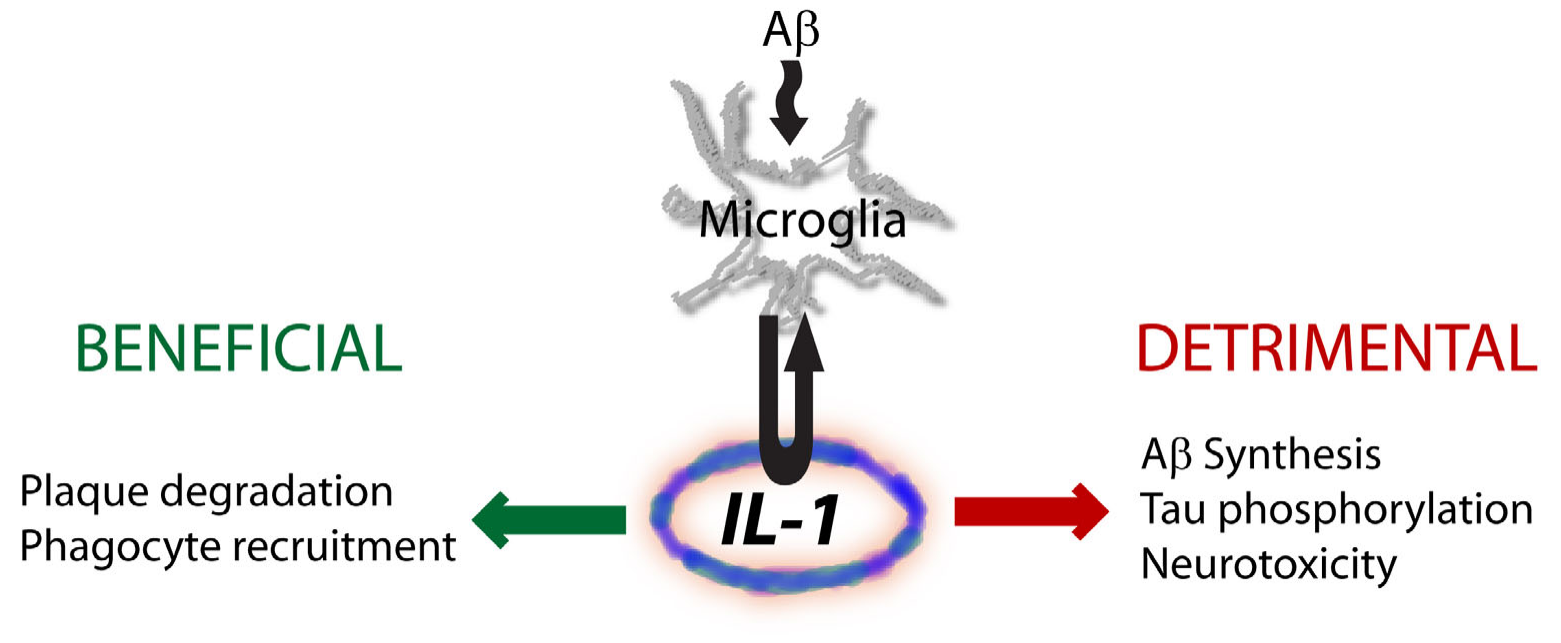
A schematic depiction of potential roles of IL-1 in AD.

At the present time our understanding of the relationship between neuroinflammation, IL-1, and AD is evolving. The downstream consequences of IL-1 elevation in AD likely involve a balance between the beneficial and detrimental functions highlighted in this review (Figure 1). Failures of recent anti-inflammatory trials in the treatment of AD may be in part explained by blockade of both beneficial and detrimental neuroinflammatory processes in the course of disease. Current findings are consistent with the idea that strategies aimed at enhancing beneficial components of neuroinflammatory pathways in chronic neurodegenerative disease may hold promise in the development of new therapies.

## Conclusion

This review highlights recent scientific studies of IL-1 activity in neuroinflammation and AD, and paints a complex picture of IL-1 function in the local tissue response of the brain to injury and disease. A clearer understanding of IL-1's role is unfolding, yielding observations of both helpful and harmful effects in experimental paradigms. This is particularly true in regard to the pathogenesis of AD, where recent observations urge a reexamination of original assumptions as to the role of IL-1 in disease. This emerging understanding of IL-1's contribution to the pathogenesis of CNS insults has identified possible roles in triggering adaptive innate immune processes during the course of chronic neurodegenerative disease. IL-1 can no longer be regarded as simply the villain in the setting of brain injury and disease, but instead might be understood as a factor that can influence the balance between beneficial and detrimental outcomes. Potentiation of such adaptive IL-1 driven responses in chronic neurodegenerative disease may provide new avenues for therapeutic intervention.

## Competing interests

The author(s) declare that they have no competing interests.

## Authors' contributions

The manuscript was written by SS as part of his doctoral thesis. MKO'B and WSTG provided historical perspectives and editorial assistance.
